# Analysis of clinical efficacy and quality of life in hypopharyngeal cancer patients undergoing laryngeal-preserving surgery

**DOI:** 10.3389/fonc.2026.1848440

**Published:** 2026-07-29

**Authors:** Jie Deng, Shengen Xu, Huajun Feng, Gang Qin

**Affiliations:** 1 Department of Otolaryngology Head and Neck Surgery, The Ziyang Central Hospital, Ziyang, China; 2 Department of Otolaryngology Head and Neck Surgery, The Affiliated Hospital of Southwest Medical University, Luzhou, China

**Keywords:** hypopharyngeal cancer, laryngeal preservation, quality of life, swallowing function, voice function

## Abstract

**Objective:**

This study analyzes the therapeutic efficacy of hypopharyngeal cancer following laryngeal function-preserving surgery, assesses the recovery of swallowing and vocal functions, provides a reference for the selection of surgical approaches and the implementation of postoperative rehabilitation training for swallowing and vocal functions, and thereby improves the therapeutic outcomes and quality of life of patients.

**Methods:**

Date from 23 patients with hypopharyngeal carcinoma who underwent laryngeal function-preserving surgery at the Department of Otorhinolaryngology Head and Neck Surgery between January 2017 and January 2024 were retrospectively analyzed in February 2025. The Kaplan-Meier method was employed to calculate the 1-, 3-, and 5-year disease-free survival (DFS) and overall survival (OS) rates. Intergroup differences were compared using the log-rank test, and the COX proportional hazards regression model was applied to identify key prognostic risk factors. Swallowing function and swallowing-related quality of life were assessed via a combination of flexible endoscopic evaluation of swallowing (FEES) and the M.D. Anderson Dysphagia Inventory (MDADI) scale. Voice function and voice-related quality of life were comprehensively evaluated using acoustic parameters (shimmer, jitter, fundamental frequency [F0]), maximum phonation time (MPT) measurements, and the 10-Item Voice Handicap Index (VHI-10). Spearman’s rank correlation analysis was used to explore the factors influencing postoperative swallowing and voice rehabilitation.

**Results:**

The 1-, 3-, and 5-year OS rates were 87.51%, 53.33%, and 42.86%, respectively, while the corresponding 1-, 3-, and 5-year DFS rates were 71.93%, 46.72%, and 42.86%. Lymph node metastasis reduced OS (*P* < 0.05). FEES showed texture-dependent pharyngeal residue, rare solid-food aspiration but frequent laryngeal penetration for semi-liquids and liquids. Vocal acoustic/aerodynamic parameters and favorable MDADI (84.17 ± 12.18) and VHI-10 (10.17 ± 8.18) scores were recorded in 23 functional-assessed patients.

**Conclusion:**

Laryngeal function-preserving surgery yields satisfactory oncological outcomes for hypopharyngeal carcinoma patients, Most patients achieved acceptable swallowing and voice function with good quality of life. This surgical approach effectively balances oncological efficacy and preserves organ function.

## 1 Introduction

Any laryngeal-preserving surgical intervention for hypopharyngeal cancer inevitably leads to partial structural loss of the hypopharynx or larynx, or alters the vocal resonance cavity, often resulting in postoperative swallowing and voice dysfunction in patients. At present, research focusing on postoperative swallowing function assessment in hypopharyngeal cancer patients who have undergone laryngeal-preserving surgery remains relatively scarce (1); additionally, existing studies lack comprehensiveness in evaluating swallowing function. A holistic assessment of swallowing function encompasses both subjective and objective evaluations, with the M.D. Anderson Dysphagia Inventory serving as the primary tool for subjective assessment (2, 3). Integrating subjective and objective assessment approaches to evaluate swallowing status in post-laryngeal-preserving surgery patients with hypopharyngeal cancer enables a more accurate measurement of their swallowing-related quality of life. For hypopharyngeal cancer patients undergoing laryngeal-preserving surgery, intraoperative resection of hypopharyngeal or laryngeal tissue inevitably impairs vocal structures, rendering postoperative voice changes an unavoidable clinical challenge (4, 5). In the realm of voice function assessment, the most commonly used metrics currently include jitter, shimmer, fundamental frequency (F0), and MPT (6). Despite the critical impact of voice function on patients’ quality of life, current research on voice function recovery in this patient population is insufficient, particularly regarding comprehensive and in-depth analysis of voice-related quality of life (7, 8). This gap to a certain extent hinders clinicians from comprehensively evaluating patients’ postoperative rehabilitation outcomes, which is not conducive to developing more targeted rehabilitation regimens to enhance patients’ quality of life. Therefore, this study conducted a retrospective analysis of medical records and follow-up data of hypopharyngeal cancer patients who underwent laryngeal-preserving surgery at the Department of Otorhinolaryngology Head and Neck Surgery, the Affiliated Hospital of Southwest Medical University, between September 2017 and February 2024. By combining subjective and objective assessment methods, we evaluated patients’ postoperative swallowing function, voice function, and related quality of life, explored the associated factors and risk factors, and aimed to provide evidence-based references for surgical option selection, treatment optimization, and postoperative rehabilitation training.

## 2 Materials and methods

### 2.1 Clinical data

#### 2.1.1 Study subjects

Clinical data of 23 eligible patients who underwent laryngeal-preserving hypopharyngeal surgery at the Affiliated Hospital of Southwest Medical University between January 2017 and January 2024 were analyzed retrospectively. All 36 enrolled patients were pathologically confirmed as hypopharyngeal squamous cell carcinoma; no other pathological subtypes (adenocarcinoma, sarcoma, etc.) were included. Primary vs recurrent tumor: All 36 patients were treatment-naive primary hypopharyngeal carcinoma (*de novo*, per primum). No patients had prior definitive radiotherapy, chemotherapy or surgical treatment for head and neck malignancy; no salvage surgery cases after radiotherapy failure were included in this cohort. Information on postoperative complications, postoperative radiotherapy status, and tumor recurrence or metastasis was also documented. Follow-up was conducted to monitor patients’ postoperative survival outcomes.

#### 2.1.2 Inclusion and exclusion criteria

##### 2.1.2.1 Inclusion criteria

①patients pathologically diagnosed with hypopharyngeal carcinoma; ②patients who underwent laryngeal-preserving hypopharyngeal surgery as the primary treatment; ③patients who could complete regular postoperative follow-up and provide complete clinical data; ④patients with a postoperative follow-up duration of at least 1 year.

##### 2.1.2.2 Exclusion criteria

①patients with distant metastasis (e.g., lung, liver, bone) detected by preoperative examination; ②patients with malignant tumors at other sites; ③patients who refused to participate in follow-up; ④patients with incomplete clinical data or unavailable postoperative follow-up data.

#### 2.1.3 Subject selection for swallowing and voice function assessment

Additional exclusion criteria for this sub-assessment included: ① Patients with mental illness or cognitive impairment who could not cooperate with follow-up and quality-of-life assessment; ② Patients with a history of pharyngeal or esophageal surgery that might affect swallowing function prior to the assessment; ③ Patients with diseases (e.g., cerebral infarction, Parkinson’s disease) that might impair the recovery of swallowing or voice function; ④ Patients who were lost to follow-up or had died.

### 2.2 Study methods

#### 2.2.1 Treatment protocol

All surgeries were performed by the same medical team from the Department of Otolaryngology-Head and Neck Surgery, Affiliated Hospital of the Southwest Medical University. Preoperatively, laryngoscopy combined with computed tomography (CT) and magnetic resonance imaging (MRI) multimodal examinations was performed to evaluate tumor characteristics and lymph node metastasis (9), which improved the accuracy of diagnosis and staging and guided the selection of surgical approaches and procedures. Transoral minimally invasive surgery was preferred for localized lesions, transcervical open laryngeal-preserving surgery was adopted for complex lesions, and total laryngectomy was performed if preservation of laryngeal function was not feasible. After tumor resection, the appropriate reconstruction method (local flap, free flap, or pedicled flap repair) was selected based on the extent of surgical defects. For cervical lymph node management, the necessity of lymph node dissection was comprehensively determined based on the imaging findings and primary lesion status. Postoperatively, personalized adjuvant radiotherapy regimens were formulated based on the pathological margin status, primary tumor size, and extent of cervical lymph node metastasis.

Preoperative MDT counseling content for laryngeal-preserving surgery candidates: Two primary curative options fully disclosed to all eligible patients: (1) Organ-preserving partial laryngopharyngeal surgery (temporary tracheostomy required in most cases); (2) Definitive radiotherapy/definitive concurrent chemoradiotherapy (CCRT), which avoids routine tracheostomy.

Counseling covered comparative advantages and disadvantages of each modality: Surgery: Higher local control rate for bulky lesions, postoperative temporary tracheostomy, swallowing/voice functional recovery training required; Definitive RT/CCRT: No tracheostomy, but higher risk of long-term radiation-induced dysphagia, xerostomia, and lower salvage surgery feasibility if recurrence occurs. Salvage surgery disclosure: All patients were fully informed that both treatment arms retained salvage surgery options for future locoregional recurrence, but salvage total laryngectomy would be the primary rescue procedure for radiotherapy failure with worse functional outcomes compared to primary organ-preserving surgery. Shared decision-making process: Final treatment modality was selected after full counseling based on tumor stage, patient age, comorbidities, functional preference, and patient’s autonomous informed consent. Revision Action: Full standardized preoperative counseling and shared decision-making description added in Methods section.

#### 2.2.2 Postoperative rehabilitation

Nasogastric feeding & swallowing rehabilitation timeline: Week 1–2 post-operation: Nasogastric tube feeding only, basic oral motor training guided by SLT daily; Tube removal threshold: No fistula, choking or aspiration on endoscopic screening; sequential food texture training (liquid → semi-liquid → solid) supervised by SLT within 1 week after extubation; Long-term rehabilitation: Outpatient SLT follow-up at 1, 3, 6, 12 months post-discharge for compensatory swallowing maneuver training. Voice rehabilitation schedule: Tracheostomy tube occlusion training 48 h before decannulation; Voice rest for 2 weeks postoperatively; progressive phonation training guided by SLT starting week 3; Resonance correction and hoarseness improvement training for patients with sustained dysphonia. Clarify SLT participation: All functional training, FEES evaluation and subjective scale assessments were completed jointly by otolaryngologists and specialized speech-language therapists; regular multi-disciplinary team (MDT) rehabilitation rounds were conducted for high-risk patients with severe aspiration.

#### 2.2.3 Postoperative follow-up

The patients’ postoperative survival status was followed up by telephone, with the follow-up period ranging from the date of surgery to January 31, 2025. Survival analysis was performed using the surgery date as the starting point and predefined events as endpoints. Overall survival was defined as death as the endpoint, while disease-free survival included death, recurrence, and metastasis as endpoints. Lost-to-follow-up cases were treated as censored data, and the survival time was calculated from the date of surgery to the last follow-up. Recovery of swallowing and voice function was assessed during outpatient follow-up.

#### 2.2.4 Assessment of swallowing function, voice function, and related quality of life

##### 2.2.4.1 Objective assessment of swallowing function (flexible endoscopic evaluation of swallowing)

A flexible endoscope was inserted through one nasal cavity, with its tip positioned at the junction of the soft palate and epiglottis, to evaluate the presence of food residue in the valleculae and pyriform sinuses before swallowing. If a residue was detected, the patient was instructed to perform three dry swallows. Subsequently, subjects were sequentially given 10 g of black biscuits, 10 mL of yogurt mixed with blue food coloring, and an equal volume of clear water were used for swallowing tests. During the examination, the primary observation focused on the retention of liquid or food in the valleculae and pyriform sinuses, which was evaluated using the Yale Pharyngeal Residue Rating Scale. The Penetration-Aspiration Scale was used to quantitatively assess the degree of food aspiration.

All FEES examiners were attending physicians of otolaryngology head and neck surgery with ≥5 years standardized swallowing endoscopy training, unified Yale Pharyngeal Residue Rating Scale and Penetration-Aspiration Scale training before study initiation;

FEES video recordings were independently graded by two blinded otolaryngologists; assessors were blinded to patient tumor stage, surgical type and radiotherapy history;

##### 2.2.4.2 Subjective assessment of swallowing function

The M.D. Anderson Dysphagia Inventory (MDADI) was used to evaluate swallowing-related quality of life (10).

##### 2.2.4.3 Objective assessment of voice function

Ling Waves software was used to record and analyze the voice data. During the recording, ambient noise was controlled below 40 dB, and the microphone was placed 30 cm away from the lips. Subjects were required to sustain the vowel/α:/continuously, with three measurements taken, and the most stable voice sample was selected to analyze basic acoustic parameters, including fundamental frequency (F0), jitter, and shimmer. For maximum phonation time (MPT) measurement, subjects were instructed to take a deep breath and then sustain the vowel/α:; the test was performed at least three times, and the sample with the longest and most stable duration was selected as the final data.

For acoustic voice analysis: Ling Waves software automatic parameter calculation was adopted to minimize artificial measurement bias; VHI-10 and MDADI questionnaires were uniformly distributed and filled out independently by patients without examiner guidance to avoid subjective bias.

##### 2.2.4.4 Subjective assessment of voice function

The 10-Item Voice Handicap Index (VHI-10) scale was used to evaluate patients’ voice function (11, 12).

#### 2.2.5 Statistical methods

Qualitative data are presented as frequencies and percentages, while quantitative data were expressed as mean ± standard deviation (x̄ ± s). The 1-, 3-, and 5-year OS and DFS rates are calculated using the Kaplan-Meier method, intergroup differences were compared using the log-rank test, prognostic factors were analyzed using the Cox proportional hazards regression model, and factors influencing voice function were explored using Spearman’s rank correlation analysis. All data analyses were performed using SPSS 22.0, and statistical significance was set at P < 0.05.

## 3 Results

### 3.1 Clinical characteristics of patients

#### 3.1.1 General clinical data

A total of 36 patients who underwent laryngeal-preserving surgery for hypopharyngeal carcinoma were enrolled in this study (**Table 1**), 35 males and 1 female. Their age range was 43–79 years, with a median age of 59 years. All patients completed the postoperative follow-up, with a follow-up duration ranging from 12 to 79 months and a median of 52 months. Thirty-one patients received postoperative radiotherapy, with an average radiation dose of 50–76 Gy. Among the 36 patients, nasogastric tubes were removed in 30 had their, yielding a tube removal rate of 83.33%. Tracheostomy tubes were removed in 26 of 36 patients, with a tube removal rate of 72.22%.

**Table 1:** Basic information and clinical characteristics of patients undergoing laryngeal function-preserving surgery for hypopharyngeal cancer.

Contextual notes	Case (%)
Gender	
Male	35(97.22)
Female	1(2.78)
Age	
≥60 years	16(44.45)
<60years	20(55.56)
Differentiation grade	
Poorly differentiated	5(13.89)
Moderately differentiated	17(47.22)
Well-differentiated	14(38.89)
Tumor location	
Pyriform sinus	24(66.67)
Posterior pharyngeal wall	12(33.34)
Postcricoid region	0(0.00)
T stage	
T1	4(11.11)
T2	18(50.00)
T3	9(25.00)
T4a	5(13.89)
N stage	
N0	13(36.11)
N1	6(16.67)
N2a	10(27.78)
N2b	5(13.89)
N2c	2(5.56)
Clinical stage	
I	1(2.78)
II	7(19.44)
III	8(22.22)
IVa	20(55.56)
Surgical type	
Transoral minimally invasive surgery	1(2.78)
Partial hypopharyngectomy	7(19.44)
Partial hypopharyngectomy + partial laryngectomy	26(72.22)
Partial hypopharyngectomy + partial esophagectomy	2(5.56)
Flap reconstruction	
Supraclavicular flap	14
Radial forearm free flap	4
Submental island flap	2
Anterolateral thigh free flap Repair with local mucosa and/or muscle tissue	1 15

#### 3.1.2 Complications, recurrence, and metastasis

Postoperative complications were observed in the 36 patients who underwent laryngeal-preserving hypopharyngeal surgery, as follows: 2 cases of pulmonary infection; 10 cases of pharyngeal fistulas (6 healed after dressing changes, anti-infection treatment, and other conservative therapies, while 4 required surgical suture repair); 2 cases of pharyngoesophageal anastomotic stenosis (swallowing function improved after surgical dilation); 1 case of necrosis of the supraclavicular pedicled flap (the pharyngeal defect was repaired with a left radial forearm free flap in a second surgery); and 1 case of massive bleeding due to major neck vascular rupture caused by surgical site infection (the patient’s family opted to discontinue treatment).

#### 3.1.3 Oncological outcomes

##### 3.1.3.1 Patient survival status

**Figure 1:**
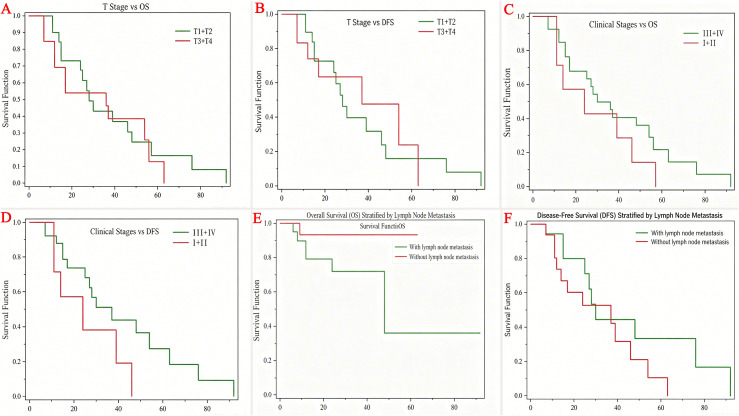
Kaplan–Meier survival curves for hypopharyngeal carcinoma patients with laryngeal function preservation. **(A)** Overall survival (OS) curves stratified by T stage: T1+T2 versus T3+T4a; **(B)** Disease-free survival (DFS) curves stratified by T stage: T1+T2 versus T3+T4a; **(C)** OS curves stratified by overall clinical stage: stage I+II versus stage III+IVa; **(D)** DFS curves stratified by overall clinical stage: stage I+II versus stage III+IVa; **(E)** OS curves stratified by lymph node metastatic status: node-negative versus node-positive patients; **(F)** DFS curves stratified by lymph node metastatic status: node-negative versus node-positive patients.

During the follow-up period, 9 patients who underwent laryngeal-preserving hypopharyngeal surgery died. The 1-year, 3-year, and 5-year overall survival rates were 87.51% (95%CI: 73.22%-95.06%), 53.33% (95%CI: 37.14%-68.32%), and 42.86% (95%CI: 27.43%-58.91%), respectively, whereas the corresponding disease-free survival rates were 71.93% (95%CI: 55.84%-84.01%), 46.72% (95%CI: 30.98%-62.87%), and 42.86% (95%CI: 27.43%-58.91%), respectively (**Table 2**). The OS and DFS rates of patients with T1+T2 stage tumors were lower than those of patients with T3+T4a stage tumors, although the difference was not statistically significant (OS:P=0.692, **Figure 1A**; DFS:P=0.429, **Figure 1B**). Patients with stage I–II tumors had higher OS and DFS rates than those with stage III–IVa tumors; however, the differences were not statistically significant. No statistically significant differences in OS and DFS were observed with respect to postoperative complications, patient age, or tumor differentiation grade (OS:P=0.608, **Figure 1C**, DFS:P=0.681, **Figure 1D**). However, the OS rate of patients with lymph node metastasis was significantly lower than that of patients without metastasis (OS:P=0.045, **Figure 1E**; DFS:P=0.538, **Figure 1F**).

**Table 2:** Survival rates of patients with hypopharyngeal cancer with preserved laryngeal function as a whole and in different groups.

Group	1-year		5-year	
	OS%	DFS%	OS%	DFS%
Patients with hypopharyngeal cancer undergoing laryngeal function preservation	87.51	71.93	42.86	42.86
T stage				
T1+T2	81.81	81.81	68.18	63.64
T3+T4a	92.86	92.86	78.57	71.43
Clinical stage				
I+II	93.75	93.75	93.75	93.75
III+IVa	80.00	80.00	60.00	50.00
Presence or absence of lymph node metastasis				
Yes	82.61	82.61	65.22	65.52
No	92.31	92.31	92.31	92.31
With or without complications				
Yes	87.50	87.50	68.75	56.25
No	85.00	85.00	80.00	80.00
Differentiation degree				
Low	83.33	83.33	66.67	66.67
Moderate + High	86.67	86.67	76.67	70.00
Age				
** ≥**60 years old	87.50	87.50	75.00	68.75
<60 years old	85.00	85.00	75.00	70.00

##### 3.1.3.2 Prognostic factor analysis

Univariate and multivariate Cox proportional hazards regression analyses (**Tables 3**, **4**) revealed that lymph node metastasis (*P* < 0.05) was an independent risk factor affecting postoperative DFS in patients.

**Table 3:** Factors influencing OS and DFS of patients undergoing surgery for hypopharyngeal cancer with preserved laryngeal function analyzed by COX univariate regression analysis.

Contextual notes	OS		DFS	
	HR (95% CI)	*P*	HR (95% CI)	*P*
(T1/T2+T3/T4a)	0.72 (0.18-2.92)	0.646	1.74 (0.60-5.0)	0.305
Age (**≥**60 years old +<60 years old)	1.03 (0.94-1.12)	0.537	1.02 (0.96-1.08)	0.516
Tumor differentiation (Low/Moderate/High differentiation)	0.75 (0.28-2.02)	0.573	0.84 (0.40-1.76)	0.648
Lymph node metastasis	0.17 (0.02-1.34)	0.092	0.44 (0.14-1.40)	0.164
Postoperative complications	0.95 (0.25-3.65)	0.936	0.45 (0.15-1.34)	0.151
Postoperative chemoradiotherapy	3.70 (0.41-33.17)	0.242	2.97 (0.35-24.5)	0.315

**Table 4:** Cox multivariate regression analysis of factors affecting OS and DFS in hypopharyngeal cancer patients undergoing laryngeal preservation surgery.

Contextual notes	OS		DFS	
	HR (95% CI)	P	HR (95% CI)	P
T (T1/T2+T3/T4a)	0.93 (0.15-5.69)	0.941	1.94 (0.45-8.36)	0.372
**≥**60 years old +<60 years old	1.07 (0.96-1.18)	0.212	1.05 (0.97-1.13)	0.238
Tumor differentiation degree (Low/Moderate/High differentiation)	0.67 (0.24-1.87)	0.450	0.82 (0.35-1.94)	0.655
Lymph node metastasis	0.13 (0.02-1.11)	0.062	0.29 (0.08-0.97)	0.044
Postoperative complications	0.68 (0.12-3.89)	0.664	0.48 (0.12-1.95)	0.302
Postoperative chemoradiotherapy	8.80 (0.50-254.81)	0.137	6.41 (0.54-76.43)	0.142

### 3.2 Swallowing and voice function

#### 3.2.1 Baseline characteristics of patients undergoing function assessment

Based on the predefined inclusion and exclusion criteria, among the 36 enrolled patients, nine died and four refused to participate in the assessments. Finally, 23 patients completed voice and swallowing quality-of-life assessment. Among these 23 patients, there were 22 males and 1 female, with an age range of 46–77 years (median: 58 years); 10 patients were over 60 years old. Of the 23 patients, 21 received postoperative radiotherapy, with a dose range of 50–76 Gy.

#### 3.2.2 Swallowing function

**Figure 2:**
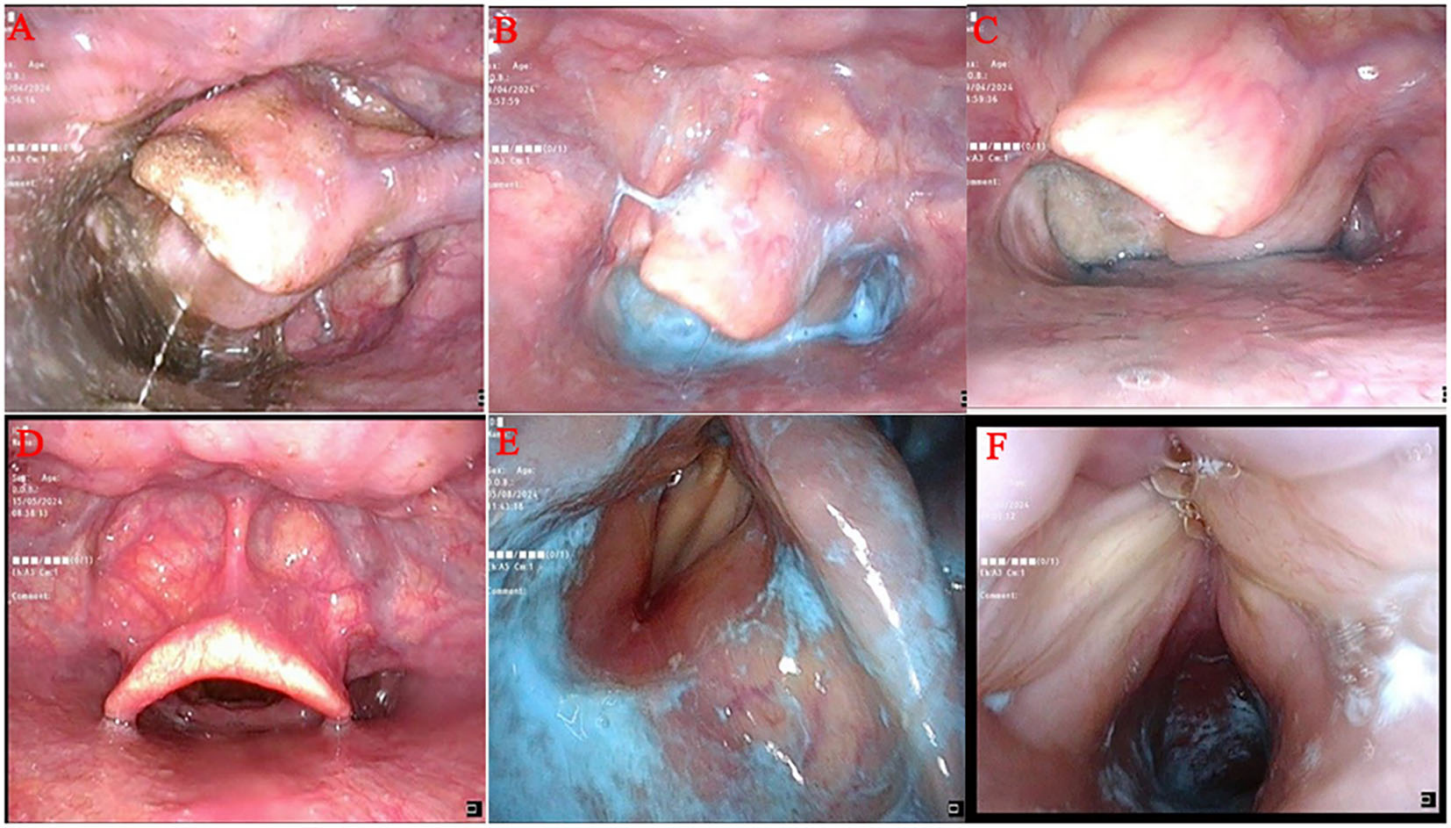
Flexible endoscopic evaluation of swallowing (FEES) images of patients after laryngeal-preserving hypopharyngeal surgery. **(A)** Pharyngeal residue observation during solid food swallowing (Yale residue Grade II–III); **(B)** Pharyngeal residue under semi-liquid food challenge; **(C)** Residue distribution after liquid food ingestion; **(D)** Normal laryngeal closure without penetration/aspiration during swallowing; **(E)** Laryngeal penetration: food material enters laryngeal vestibule above vocal folds; **(F)** Aspiration: food passes through vocal folds into trachea. All tests used dyed food for clear visualization of residue and penetration-aspiration events.

Nasogastric tubes were removed in all 23 patients, resulting in a 100% tube removal rate (23/23). The results of flexible endoscopic evaluation of swallowing in these 23 patients are presented as follows: pharyngeal residue (**Table 5**, **Figures 2A–C**), aspiration status (**Table 6**, **Figures 2D–F**), and laryngeal penetration rates when swallowing solid, semi-liquid, and liquid foods (**Tables 5**, **6**).

**Table 5:** Yale pharyngeal residue severity rating scale (YPR - SRS) (n = 23).

Group	Grade I	Grade II	Grade III	Grade IV	Grade V
Solid	0	11(47.83%)	6(26.09%)	6(26.09%)0	0
Semi-liquid	0	13(56.52)	5(21.74%)	5(21.74%)	0
Liquid	4(17.39%)	15(65.22)	4(17.39%)	0	0

**Table 6:** Laryngeal aspiration conditions (n = 23).

Group	Laryngeal penetration	Laryngeal penetration rate	Aspirationn	Aspiration rate
Solid	6	26.09%	0	0
Semi-liquid	18	78.26%	3	13.04%
Liquid	17	73.91%	4	17.39%

#### 3.2.3 Voice function

Among the 23 patients, the mean values of voice acoustic parameters were as follows: fundamental frequency (F0) = 125.37 ± 22.63 Hz; jitter = 4.35 ± 5.46%; shimmer = 18.83 ± 14.52%. For the aerodynamic parameters, the mean maximum phonation time (MPT) was 6.65 ± 1.43 seconds. Spearman’s correlation analysis was performed between the voice acoustic parameters, aerodynamic assessment results, and clinical characteristics (including patient age, radiotherapy status, and T stage). The results showed a significant negative correlation between the MPT and age (correlation coefficient R = -0.579, *P* < 0.05) (**Table 7**).

**Table 7:** Spearman correlation analysis of voice function indicators and clinical data in patients after laryngeal function-preserving hypopharyngeal surgery (n = 23).

Group	Age		Postoperative radiotherapy		T stage	
	*R*	*P*	*R*	*P*	*R*	*P*
F0	0.134	0.541	-0.273	0.207	0.083	0.707
Jitter	0.222	0.308	-0.480	0.827	0.068	0.758
Shimmer	-0.110	0.617	-0.273	0.207	-0.112	0.609
MPT	-0.579	0.040	-0.362	0.090	0.290	0.179

### 3.3 Analysis of swallowing- and voice-related quality of life

Among the 23 patients, the mean total score of the M.D. Anderson Dysphagia Inventory (MDADI) was 84.17 ± 12.18, which was substantially higher than 60 points, indicating that patients were relatively satisfied with their swallowing-related quality of life. The mean scores of each MDADI subdomain were as follows: Functional = 21.04 ± 3.04; Physical = 33.52 ± 5.49; Emotional = 25.70 ± 2.82. The mean total score of the 10-Item Voice Handicap Index (VHI-10) was 10.17 ± 8.18. A total score of less than 11 points indicated that patients had a high recognition of their own voice status and believed that voice disorders did not have a serious negative impact on their quality of life. The mean scores of each VHI-10 subdomain were as follows: Functional = 6.00 ± 4.85; Physical = 2.74 ± 2.42; Emotional = 1.39 ± 0.99 (**Table 8**).

**Table 8:** Scores of MDADI and VHI - 10 scales.

Group	MDADI scale score (x̄±s)	VHI-10 scale score (x̄±s)
Total score	84.17±12.18	10.17±8.18
Physical	33.52±5.49	2.74±2.42
Functional	21.04±3.04	6.00±4.85
Emotional	25.70±2.82	1.39±0.99

## 4 Discussion

Primary surgical approaches for laryngeal function preservation in hypopharyngeal carcinoma include transoral minimally invasive surgery (TOMIS), partial hypopharyngectomy, and partial laryngectomy. TOMIS is indicated in carefully selected patients with early-stage hypopharyngeal carcinoma (characterized by small tumor foci and easily accessible lesions), with available options such as transoral CO₂ laser microsurgery (TLM), transoral radiofrequency coblation surgery (TRCS), and transoral videolaryngoscopic surgery (TOVS) (13–16). With advancements in medical technology, transoral robotic surgery (TORS) has been increasingly applied in clinical practice (17–19).

Partial hypopharyngectomy is a commonly used laryngeal-sparing procedure, however, its indications must be strictly controlled and primarily limited to early-stage tumors. When hypopharyngeal carcinoma invades the laryngeal cavity, partial hypopharyngectomy combined with partial laryngectomy is preferred (20).

A retrospective study analyzed the efficacy and prognosis of 93 patients with malignant hypopharyngeal tumors and found that the 5-year overall survival rate was 49.1% in 53 patients who underwent laryngeal-sparing surgery, compared with 43.0% in 40 patients who underwent total laryngectomy (21). Another retrospective study confirmed that the 5-year OS of laryngeal-sparing surgery for hypopharyngeal carcinoma was comparable to that of total laryngectomy (22). Consistent with these findings, the 5-year OS rate in our study was 42.9%, indicating that laryngeal-sparing surgery still achieved oncological outcomes equivalent to those of total laryngectomy.

The prognosis of hypopharyngeal carcinoma is influenced by multiple factors, including age, lymph node metastasis status, clinical stage, tumor differentiation, anatomical location, and distant metastasis. For early-stage disease, comprehensive treatment (such as surgery combined with radiotherapy) yields a relatively high 5-year survival rate of 50-70%. In contrast, advanced-stage patients often present with regional or distant metastasis, with the 5-year survival rate dropping below 30% (23). A previous study identified ≥3 metastatic lymph nodes as an important predictor of poor prognosis (24). Univariate and multivariate analyses of factors affecting OS and disease-free survival revealed that patients with cervical lymph node metastasis had significantly lower OS than those without cervical lymph node metastasis, which is consistent with previous findings. No statistically significant differences were observed in the effects of age, clinical stage, or tumor differentiation on OS and DFS, which may be attributed to the small sample size of our study.

Li et al. noted that surgery-based comprehensive treatment could preserve good swallowing function without compromising tumor control (25). Their study identified that independent factors affecting OS and DFS included surgical defect size, local recurrence, and distant metastasis; independent factors for swallowing function included pharyngeal fistula and local recurrence; and independent factors for quality of life included clinical stage, local recurrence, nasogastric tube removal status, and feeding tube placement.

Patients undergoing laryngeal-sparing surgery for hypopharyngeal carcinoma require partial hypopharyngectomy or a combined hypopharyngeal and partial laryngeal resection. Postoperatively, patient may experience temporary dysphagia and face the risk of aspiration pneumonia. Variations exist in the reported postoperative aspiration and reoperation rates across different institutions. Balaji A et al. found that the incidence of dysphagia was higher in patients who underwent total laryngectomy combined with pharyngectomy than in those who underwent laryngeal-sparing pharyngectomy (26). Moreover, dysphagia severity was positively correlated with the extent of resection; patients with small, non-laryngeal lesions had better postoperative swallowing function. Maximizing the preservation of laryngeal and hypopharyngeal mucosa helps maintain swallowing function.

Our flexible endoscopic evaluation of swallowing results showed the following: for solid food, residue was predominantly Grade II (47.83%) and Grade III/IV (26.09% each); for semi-liquid food, Grade II (56.52%) and Grade III/IV (21.74% each); and for liquid food, Grade I (no residue, 17.39%), Grade II (65.22%), and Grade III (17.39%). Regarding aspiration status: no aspiration occurred with solid food (0.00%); and the aspiration rate was 8.69% with semi-liquid food and 17.39% with liquid food. The laryngeal penetration rates for solid, semi-liquid, and liquid foods were 26.09%, 78.26%, and 73.91%, respectively. Postoperatively, the patients had varying degrees of food residue with all food consistencies, which was predominantly minimal to mild (Grade I-II) according to the Yale Pharyngeal Residue Severity Scale. Laryngeal penetration rates were low for solid food but high for semi-liquid and liquid food. Aspiration was also more common with the latter two, although the incidence was low (semi-liquid: 13.04%; liquid: 17.39%). The FEES results were consistent with those of the M.D. Anderson Dysphagia Inventory, indicating good recovery of swallowing function in most patients. MDADI scores were positively correlated with satisfaction with swallowing-related quality of life; the mean total MDADI score in our study was 84.17 ± 12.18 (> 80 points), suggesting high patient satisfaction.

Combined hypopharyngeal and partial laryngeal resection impairs vocal function due to laryngeal tissue loss. Although reconstructed glottic structures increase vibration diversity, sound source quality decreases, resulting in hoarseness. Patients who underwent only partial hypopharyngectomy may also experience voice quality changes due to resonance cavity alterations. Postoperative radiotherapy can reduce jitter and shimmer values, because radiotherapy induces laryngeal tissue fibrosis and reduces involuntary movements, thereby decreasing the variation rate between adjacent cycles (27). The mean values of vocal acoustic parameters in our study were as follows: fundamental frequency (F0) = 125.37 ± 22.63 Hz, jitter = 4.35 ± 5.46%, shimmer = 18.83 ± 14.52%. No correlations were found between F0, jitter, shimmer, MPT and T stage or radiotherapy, consistent with the findings of Makeieff et al. (28).

Naturally, surgical heterogeneity is acknowledged as a limitation of this study. Due to the small sample size of subgroups receiving individual surgical approaches, stratified subgroup analyses comparing different surgical modalities were not performed in the present research. Large-sample stratified studies will be conducted in the future to eliminate confounding effects caused by surgical heterogeneity. Survivorship bias may overestimate postoperative swallowing and voice recovery outcomes, and the functional findings of this study cannot be generalized to all patients with hypopharyngeal carcinoma who underwent laryngeal-preserving surgery, particularly those with poor prognosis who died during follow-up. Flexible endoscopic evaluation of swallowing has inherent limitations. Future investigations may combine videofluoroscopic swallowing study with repeated serial endoscopic examinations during follow-up to enable more comprehensive swallowing function assessment. The absence of a concurrent internal control group prevents direct horizontal comparisons of survival and functional parameters across different treatment regimens. We plan to carry out a prospective matched case-control study with a total laryngectomy control group to compare oncological efficacy and various functional indicators between the two cohorts.

## 5 Conclusion

Laryngeal function-preserving surgery for hypopharyngeal carcinoma has yielded satisfactory oncological outcomes. Patients achieved favorable scores in subjective quality of life related to swallowing and voice function, which is consistent with the results of objective functional assessments. Clinically, treatment regimens should be formulated based on tumor location, patient physical status, and personal willingness. On the premise of accurate and radical tumor resection, most normal laryngeal tissues should be preserved and repaired, which not only ensures patient survival rates, but also facilitates the favorable recovery of swallowing and voice functions, and further improves patients’ quality of life. This single-center small-sample study limits the external generalizability of its conclusions. Large-sample, multicenter prospective studies are needed in the future to verify the findings of the present research.

## Data Availability

The raw data supporting the conclusions of this article will be made available by the authors, without undue reservation.
